# Clinical outcomes of combined Preserflo Microshunt implantation and cataract surgery in open-angle glaucoma patients

**DOI:** 10.1038/s41598-021-95217-x

**Published:** 2021-08-02

**Authors:** José M. Martínez-de-la-Casa, Federico Saenz-Francés, Laura Morales-Fernandez, Lucia Perucho, Carmen Mendez, Ana Fernandez-Vidal, Sofía Garcia-Saenz, Ruben Sanchez-Jean, Julian García-Feijoo

**Affiliations:** Ophthalmology Unit, Department of Ophthalmology and ORL, Faculty of Medicine, Hospital Clinico San-Carlos, Universidad Complutense de Madrid, Instituto de Investigación Sanitaria del Hospital Clínico San-Carlos (IdISSC), Calle del Prof Martín Lagos, s/n, 28040 Madrid, Spain

**Keywords:** Glaucoma, Optic nerve diseases

## Abstract

To assess the effectiveness and safety of the Preserflo Microshunt (PMS) implantation combined with cataract surgery in open-angle glaucoma (OAG) patients. Retrospective, open-label study conducted on insufficiently controlled OAG patients, who underwent a PMS implant procedure with mitomycin-C 0.2%, either alone or in combination with cataract surgery, and were followed for at least 12 months. Success was defined as an intraocular pressure (IOP) ≤ 18 mmHg and a reduction of at least 20% without (complete) or with (qualified) hypotensive medication. Fifty-eight eyes were included in the study, 35 eyes underwent PMS alone and 23 underwent PMS + Phaco. In the overall study sample, mean IOP was significantly lowered from 21.5 ± 3.3 mmHg at baseline to 14.6 ± 3.5 mmHg at month 12 (p < 0.0001). The IOP was significantly reduced in both groups; p < 0.0001 each, respectively. Ocular hypotensive medication was significantly reduced (p < 0.0001) in both groups. No significant differences were observed in IOP lowering or medication reduction between groups. At month 12, 62.1% eyes were considered as complete success and 82.8% eyes as qualified success. The most common adverse events were device close-to-endothelium, conjunctival fibrosis, and wound leakage. PMS, either alone or in combination with phacoemulsification, may be considered as a valuable option for treating OAG patients.

## Introduction

Glaucoma is a chronic and progressive optic neuropathy that, in advance stages, has a significant impact on patient’s quality of life^1,2^. Different treatment strategies, focused mainly on lowering intraocular pressure (IOP), are currently available for addressing its therapeutic management^3^.

Despite trabeculectomy and drainage devices have shown to be effective for lowering IOP^4^, they may be associated with different complications, some of them severe, that may lead to visual impairment^5^. This has led to the search for safer surgical alternatives, but with similar efficacy.

Under the term minimally invasive glaucoma surgery (MIGS) different devices and surgical procedures, which modulate aqueous humor outflow facility via one of several routes, have emerged^6^. Preserflo Microshunt (PMS, Santen Inc., Miami, FL; formerly known as the InnFocus Microshunt) is a new ab-externo and subconjunctival MIGS device designed for treating patients with early to advanced open-angle glaucoma (OAG).

Unlike other MIGS devices, such as the Xen 45 Gel Stent (Allergan, Irvine, California, USA), which are implanted with an ab-interno approach, PMS is inserted ab externo^7^.

The PMS is a tube composed of polystyrene‐block‐isobutylene‐block‐styrene (SIBS), a biostable thermoplastic elastomer that has demonstrated biocompatibility and long-term stability^8^. SIBS has been associated with minimal inflammation or encapsulation, especially when compared with other implant materials, such as silicone rubber^9^.

The currently available device is an 8.5-mm long SIBS tube with an outer diameter of 350 μm and a lumen diameter of 70 μm^9^. This device has a 1.1-mm wingspan ‘fin’, located approximately halfway down the tube to prevent leakage around the tube and to fix the tube to the sclera^7,9^.

Currently available scientific evidence has mainly evaluated the effectiveness of PMS in a clinical trial setting^10–14^, but there is only scarce evidence about its effectiveness in the real-world clinical practice.

Real-world studies represent a strategy for providing complementary evidence to that provided by randomized controlled trials (RCT). Although RCT is a well known method for obtaining high-quality evidence about efficacy and safety of medical interventions, the extremely strict inclusion/exclusion criteria may exclude most of the patients seen in clinical practice, and therefore, their study populations differ significantly from that found in daily practice^15^. Data from real-world studies, provide information about the effectiveness and safety of a medical intervention in large populations^16^.

The purpose of the current paper is to evaluate the efficacy and safety of PMS implantation combined with cataract surgery in patients with open-angle glaucoma. Additionally, we compared the clinical outcomes between PMS alone and in combined surgery.

## Materials and methods

### Design

This was a retrospective, two centers (Hospital Clínico San Carlos and Clínica Martínez de la Casa Matilla), open-label study conducted in Madrid (Spain). The study protocol adhered to the tenets of the Declaration of Helsinki and was approved by the Ethics Committee of the Hospital Clinico San Carlos. Written informed consent was obtained from all participants before surgery. Good Clinical Practices were followed.

### Study participants

This study included patients with insufficiently controlled OAG who underwent a PMS implant procedure, either alone or in combination with cataract surgery, and had at least a minimum postoperative follow-up period of 12 months. Patients with any type of glaucoma other than OAG, conjunctival scarring, phacodonesis, or any condition that in the surgeon’s opinion might compromise the procedure outcomes, were excluded.

Patients with cataract (score ≥ 2, according to the lens Opacities Classification System III^17^) or significant cataract-related symptoms were selected for combined surgery (PMS plus phacoemulsification).

Patients were instructed to withdraw topical and systemic ocular hypotensive medications on the day of surgery.

### Surgical technique

The PMS was provided in a sterile packaged kit containing a 3-mm scleral marker, a 1-mm triangular-blade knife, 3 LASIK Shields (EYETEC, Antwerp, Belgium), a marker pen, and a 25-gauge needle.

All surgical procedures were performed under local anesthesia by the same surgeon (JMC). After skin disinfection, a conjunctival peritomy was performed approximately 3–4 mm in length between the superior rectus muscles and either the medial or lateral rectus. By using a 25G cannula, 1–2 ml of lidocaine 2% were injected through a small conjunctival eyelet at the beginning of the peritomy with the aim of creating a blister, which facilitates subsequent conjunctival dissection. After removal from the sterile package, the PMS was rinsed with balanced sterile saline (BSS) solution.

Once Tenon’s capsule was dissected, a deep pocket was created, by using blunt scissors, between the rectus muscles to approximately the equator. Bipolar cautery was used to achieve light hemostasis, as needed.

Topical Mitomycin C (MMC) (0.2 mg/mL) was placed into the subconjunctival space using three LASIK Shields for 2 min.

After marking the sclera 3 mm from the limbus with the scleral marker, a 1-mm-wide, 1–2-mm-long, shallow scleral pocket was made 3 mm posterior to and towards the limbus with a triangular-bladed knife. A 25-gauge needle was then passed through the scleral pocket into the anterior chamber, remaining parallel to the iris plane to decrease the risk of corneal endothelial cell loss, and then retracted, thereby creating a dissected tunnel. The PMS was threaded bevel-up through the needle tunnel with forceps, and the 1.1-mm wingspan planar fins of the device were wedged into the 1-mm scleral pocket. A 23 thin-wall cannula was used to flush the PMS from the distal end of the tube. The flow of aqueous humor through the lumen of the PMS was visually confirmed by the formation of a droplet on the distal tip of the device. The distal end of the device was then tucked beneath the conjunctiva and Tenon’s capsule, followed by separate closure of the conjunctiva and Tenon’s layer with 9-0 vicryl or with 9-0 Nylon sutures. The site was checked for bleb leaks.

In patients who underwent cataract surgery, phacoemulsification was performed first with a standard technique and PMS implantation followed as previously described.

Postoperative care included topical steroids and antibiotics. Steroid eye drops were slowly tapered over 3 months.

Post-operatively, topical therapy was restarted if according to the surgeon’s opinion, IOP was not adequate.

### Study visits

The study protocol included a baseline visit (performed within 1 month before surgery) and 4 postoperative visits. Follow-up visits were performed at week 1 (± 1 day); months 1 and 3 (± 15 days); and months 6 and 12 (± 30 days).

At baseline demographic characteristics, IOP, best corrected visual acuity (BCVA), slit-lamp examination of the anterior segment, dilated funduscopic examination, and computerized visual field (Octopus, Haag-Streit AG, formerly Interzeag AG, Schlieren, Switzerland) were collected.

Follow-up visits included a review of the medical history, slit-lamp examination of the anterior segment with dilated pupils, IOP, and dilated funduscopic examination. BCVA was measured at baseline and at the last follow-up visit (12 months).

### Study groups

The study sample was divided in two groups: Group 1 (PMS), eyes underwent PMS implant alone; Group 2 (PMS + Phaco), eyes underwent PMS implant combined with phacoemulsification surgery.

### Definitions

Complete success was defined as a month-12 IOP ≤ 18 mmHg and an IOP reduction ≥ 20% compared to baseline, without any hypotensive medication at month-12 visit.

Qualified success was defined as a month-12 IOP ≤ 18 mmHg and an IOP reduction ≥ 20% compared to baseline, with topical hypotensive medication at month-12 visit.

Patients with an IOP < 6 mmHg for more than 2 consecutive visits, those who needed further glaucoma surgery, or those who had surgery for complications were also considered as failure.

### Outcomes

The primary endpoints were the mean change in IOP and number of hypotensive medications from baseline to month-12.

Secondary end-points included the proportion of patients considered as complete or qualified success.

Safety analysis included incidence of adverse events.

### Statistical analysis

A standard statistical analysis was performed using SPSS Statistics (version 24; IBM, Armonk, NY, USA).

Data was tested for normal distribution using a Kolmogorov Smirnov test.

Variables with a normal distribution were expressed as mean ± SD and compared using repeated measures ANOVA and the Greenhouse–Geisser correction test.

The Mann–Whitney *U* test was used in the evaluation of the baseline clinical and demographic parameters between PMS and PMS + Phaco eyes. The Mann–Whitney *U* test was also used in the evaluation of the changes in IOP and number of ocular hypotensive medications from baseline to month 12 between PMS + Phaco groups.

Complete success survival rates were plotted for Study groups using Kaplan–Meier analysis and were compared using a log-rank test.

Categorical variables were expressed as numbers (percentages) and compared with a Fisher exact test or Chi-squared test as appropriate.

A p value of less than 0.05 was considered significant, and all tests were 2 tailed.

### Ethics declaration

All procedures performed in studies involving human participants were in accordance with the ethical standards of the institutional and/or national research committee and with the 1964 Helsinki declaration and its later amendments or comparable ethical standards. The local ethics committee waived the need for written informed consent of the participants for the study.

### Ethics approval

This study was approved by the local ethics committees and was performed with the principles of the Declaration of Helsinki.

## Results

Fifty-eight eyes fulfilled the inclusion and exclusion criteria, 35 (60.3%) eyes underwent PMS and 23 (39.7%) ones underwent PMS + Phaco.

In the overall study sample mean age was 70.8 ± 6.6 years, with no differences between PMS (70.4 ± 6.8 years) and PMS + Phaco (71.5 ± 6.1 years), p = 0.5296 Table 1 summarizes the main baseline demographic and clinical characteristics.

**Table 1:** Main demographic and clinical characteristics of the study sample. p values were calculated with Mann–Whitney *U* test.

Variable	Overall	PMS	PMS + Phaco	p
**Age, years**				
Mean ± SD	70.8 ± 6.6	70.4 ± 6.8	71.5 ± 6.1	0.529
**Sex, n (%)**				
Women	30 (51.7%)	19 (54.3%)	11 (47.8%)	0.789^a^
Men	28 (48.3%)	16 (45.7%)	12 (52.2%)	
**IOP, mmHg**				
Mean ± SD	21.5 ± 3.3	21.3 ± 3.2	21.7 ± 3.5	0.761
**NOHM**				
Mean ± SD	2.3 ± 0.5	2.3 ± 0.5	2.4 ± 0.5	0.581
**BCVA**				
Mean ± SD	0.5 ± 0.2	0.6 ± 0.1	0.4 ± 0.1	< 0.001
**MD, dB**				
Mean ± SD	6.2 ± 3.9	− 6.0 ± 3.7	− 6.5 ± 4.2	0.690
**Glaucoma type, n (%)**				
OAG	45 (77.6%)	27 (77.1%)	18 (78.3%)	0.973^b^
PEX	10 (17.2%)	6 (17.1%)	4 (17.4%)	
PIG	3 (5.2%)	2 (5.7%)	1 (4.3%)	

^a^Fisher exact test.

^b^Chi-squared test.

*N* number of eyes, *SD* standard deviation, *IOP* intraocular pressure, *NOHM* number of ocular hypotensive medications, *PMS* Preserflo Microshunt, *BCVA* best corrected visual acuity, *MD* mean defect, *OAG* open-angle glaucoma, *PEX* pseudoexfoliative glaucoma, *PIG* pigmentary glaucoma.

With the exception of the BCVA (p < 0.05), there were no significant differences in any of the baseline variables between study groups.

**Figure 1:**
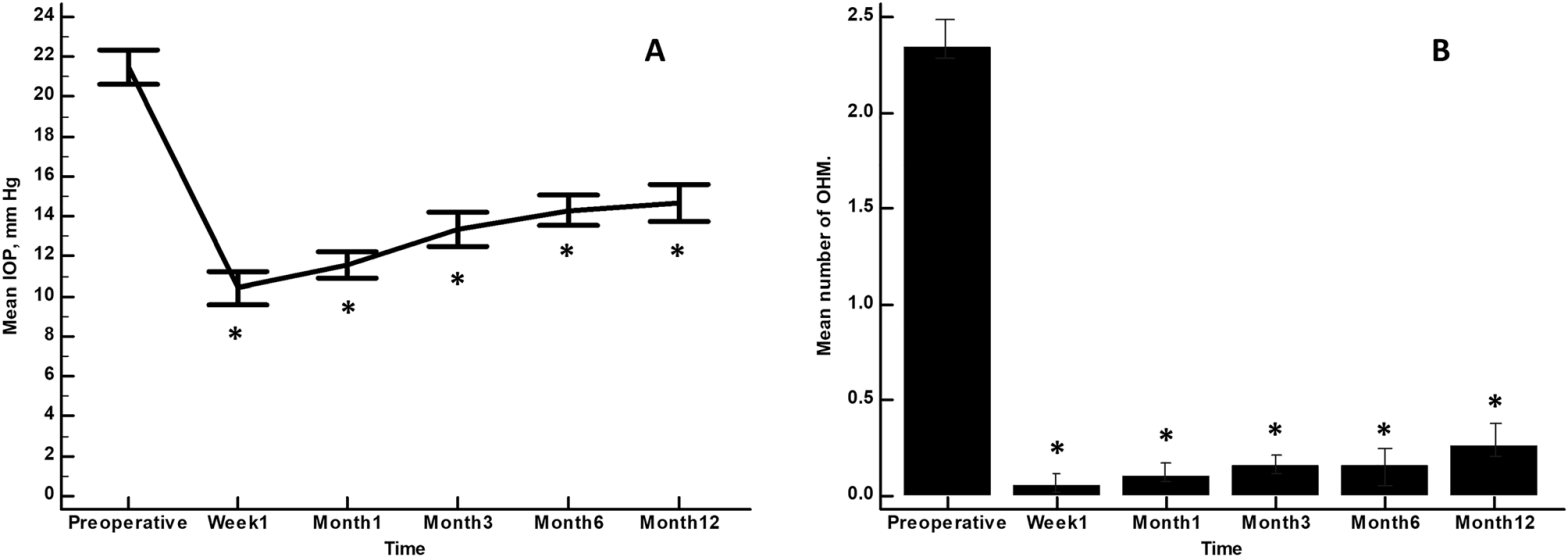
Overview of the mean intraocular pressure (**A**) and number of ocular hypotensive medications (**B**) throughout the study. Vertical bars represent 95% confidence interval. *p < 0.0001 as compared to baseline (repeated measures ANOVA and the Greenhouse–Geisser correction).

In the overall study sample, mean IOP was significantly lowered from 21.5 ± 3.3 mmHg at baseline to 10.4 ± 3.1 mmHg; 11.6 ± 2.5 mmHg; 13.3 ± 3.3 mmHg; 14.3 ± 3.0 mmHg, and 14.6 ± 3.5 mmHg at week 1, month 1, month 3, month 6, and month 12 of follow-up, respectively (p < 0.0001 each) (Fig. 1A).

Similarly, mean number of ocular hypotensive medications was significantly reduced from 2.3 ± 0.5 at baseline to 0.2 ± 0.5 at month 12 (p < 0.0001) (Fig. 1B).

**Figure 2:**
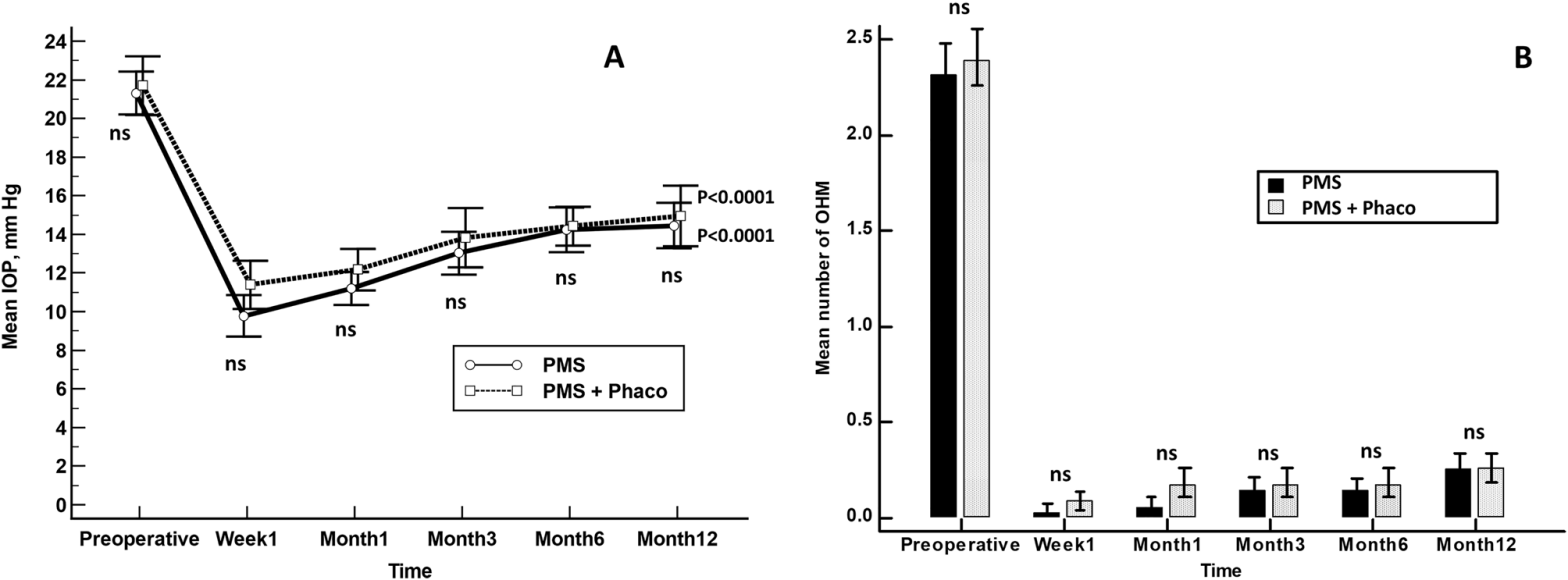
A comparison of mean intraocular pressure (IOP) (**A**) and number of ocular hypotensive medications (**B**) between PMS and PMS + Phaco groups. Statistical significance between groups was determined using the Mann–Whitney *U* test. p < 0.0001 as compared to baseline (repeated measures ANOVA and the Greenhouse–Geisser correction). *IOP* intraocular pressure, *OHM* ocular hypotensive medications, *ns* not significant.

Mean IOP was significantly lowered from 21.3 ± 3.2 mmHg and 21.7 ± 3.5 mmHg to 14.4 ± 3.4 mmHg and 14.9 ± 3.6 mmHg in the PMS and PMS + Phaco groups, respectively; p < 0.0001 each (Fig. 2A). With the exception of the day 1 (significantly lower in the PMS group), there were no statistically significant differences at any of the measured IOP time points between the PMS and PMS + Phaco groups (Fig. 2A).

There was no significant difference in the mean reduction of hypotensive medications between PMS [mean difference − 2.1 ± 0.7; 95% CI − 2.3 to − 1.8, p < 0.0001)] and PMS + Phaco [mean difference − 2.1 ± 0.6; 95% CI − 2.4 to − 1.9, p < 0.0001)], p = 0.7613 (Mann–Whitney *U* test) (Fig. 2B).

**Figure 3:**
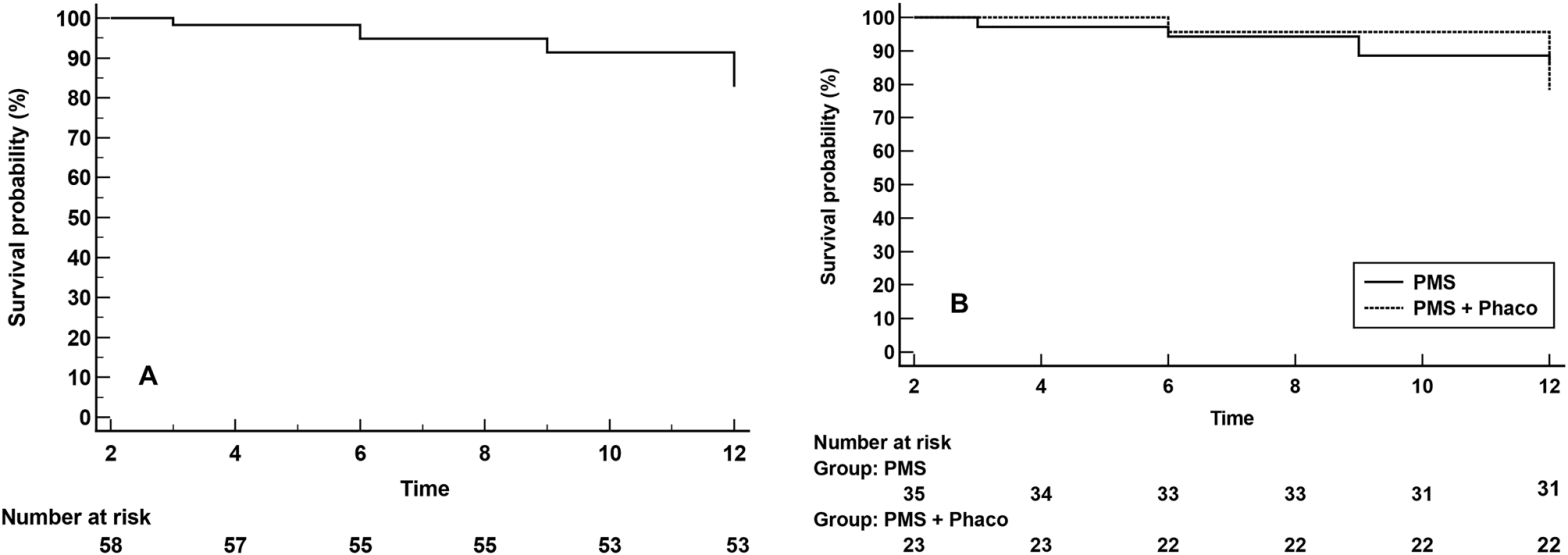
Kaplan–Meier survival curves for failure. (**A**) In the overall study sample. Failure occurred in 10 (17.2%) eyes. (**B**) In eyes treated with PMS alone (solid line) and combined surgery (PMS + Phaco) (dotted line). Failure occurred in 5 (14.3%) PMS-treated eyes and 5 (21.7%) PMS + Phaco-treated eyes. Mean hazard ratio (HR) 0.67, 95% confidence interval 0.18 to 2.44); p = 0.5440.

At month 12, 36 (62.1%) eyes were considered as complete success and 48 (82.8%) eyes as qualified success (Fig. 3A). Complete success rate was 68.6% (24/35 eyes) and 52.2% (12/23 eyes) in the PMS and PMS + Phaco groups, respectively, p = 0.1862 (Table 2).

**Table 2:** Overview of the proportion of patients who were classified as complete or qualified success over the course of the study follow-up.

	Overall, n (%)		PMS, n (%)		PMS + Phaco, N (%)		P complete	P qualified
	Complete	Qualified	Complete	Qualified	Complete	Qualified		
Week 1	55 (94.8)	58 (100.0)	34 (97.1)	35 (100.0)	21 (91.3)	23 (100.0)	0.3347	1.000
Month 1	53 (91.4)	55 (94.8)	33 (94.3)	33 (94.3)	20 (87.0)	22 (95.7)	0.3361	0.8149
Month 3	46 (79.3)	50 (86.2)	28 (80.0)	30 (85.7)	18 (78.3)	20 (87.0)	0.8768	0.8892
Month 6	41 (70.7)	48 (82.8)	25 (71.4)	29 (82.9)	16 (69.6)	19 (82.6)	0.8839	0.9766
Month 12	36 (62.1)	48 (82.8)	24 (68.6)	30 (85.7)	12 (52.2)	18 (78.3)	0.2119	0.4693

*PMS* Preserflo Microshunt.

Kaplan–Meier survival analysis did not find any difference in the success rate between PMS and PMS + Phaco groups (mean hazard ratio 0.67; 95% CI 0.18–2.44, p = 0.5440) (Fig. 3B).

Regarding safety, the most common adverse events were device proximity to the endothelium (5, 8.6%), conjunctical fibrosis (5, 8.6%) and bleab leaks (3, 5.2%) (Table 3). All the adverse events were mild in severity and were successfully resolved with medical treatment. No sight threatening complications were observed.

**Table 3:** Adverse events observed or reported during the study follow-up.

Adverse events, n (%)	Overall	PMS	PMS + Phaco	p
Hypotony*	1 (1.7)	1 (2.8)	0 (0.0)	0.4223
Seidel	3 (5.2)	1 (2.8)	2 (8.7)	0.3237
Device obstruction	2 (3.5)	2 (5.7)	0 (0.0)	0.2480
Choroidal detachment	2 (3.5)	1 (2.8)	1 (4.3)	0.7597
Conjunctival fibrosis	5 (8.6)	3 (8.6)	2 (8.7)	0.9895
Hyphema	2 (3.5)	1 (2.8)	1 (4.3)	0.7597
Device close-to-endothelium	5 (8.6)	3 (8.6)	2 (8.7)	0.9895
Implant extrusion	1 (1.7)	1 (2.8)	0 (0.0)	0.4223

*Defined as an intraocular pressure < 6 mmHg during at least 4 weeks.

*PMS* Preserflo Microshunt.

## Discussion

The results of the current study found that PMS was effective for lowering IOP and reducing the number of hypotensive medications in patients with OAG over a 12-months period.

Additionally, we also observed no differences between PMS alone or in combination with cataract surgery in either IOP lowering or reduction in number of hypotensive medications.

Finally, it should be highlighted the proportion of eyes classified as success, with 36 (62.1%) eyes considered as complete success and 48 (82.8%) eyes as qualified success.

The good IOP lowering effect observed in our study was not unexpected and did not significantly differ from that previously reported.

Batlle et al.^11^, in an observational study, assessed the efficacy and safety of PMS, alone or in combination with phacoemulsification, using intraoperatively Mitomycin C, in 23 eyes with uncontrolled glaucoma. Mean preoperative IOP was significantly lowered from 23.8 ± 5.3 to 10.7 ± 2.8 mmHg at year-1 (mean difference − 13.1 ± 4.2 mmHg; 95% CI − 15.6 to − 10.6 mmHg, p < 0.0001), with a qualified success rate (IOP ≤ 14 mmHg and IOP reduction ≥ 20%) of 100% at year-1. Additionally, the mean number of ocular hypotensive medications was reduced from 2.4 ± 0.9 at baseline to 0.3 ± 0.8 drugs at year-1^11^.

The IOP lowering effect reported by Batlle et al.^11^ is higher than that observed in our study (− 6.8 ± 4.5 mmHg; 95% CI − 9.8 to − 5.6 mmHg, p < 0.0001), although the reduction of the ocular hypotensive medication was similar (− 2.1 ± 0.7; 95% CI − 2.3 to − 1.9, p < 0.0001). The difference in IOP lowering may be explained by the different concentration of MMC (0.4% in the Batlle et al. study versus 0.2% in our study).

Similarly, Scheres et al.^12^, in a retrospective study, which included 14 eyes who underwent PMS implantation, reported that mean IOP was significantly decreased from 20.1 ± 5.0 mmHg at baseline to 13.3 ± 2.9 at month-12 (mean difference − 6.8 ± 4.1 mmHg, 95% CI − 10.0 to − 3.6 mmHg, p < 0.0001).

We found a slightly greater proportion of complete success (62.1% vs 58.0%, respectively) and qualified success (82.8% vs 79.0%, respectively) than that reported by Scheres et al. at month 12, although our sample had a higher baseline IOP^12^.

The long-term effectiveness of PMS has been evaluated by Batlle et al. in a cohort of POAG patients, who underwent PMS implantation with adjunctive MMC 0.4 mg/ml^13^. Mean IOP reduction from baseline to year-4 was − 11.0 ± 5.6 mmHg; 95% CI − 14.5 to − 7.5 mmHg, p < 0.0001^13^.

The results of a European multicenter study of patients with glaucoma, who underwent PMS implant with MMC 0.4 mg/mL, found a mean IOP reduction of − 8.4 ± 3.4 mmHg; 95% CI − 9.9 to − 6.9 mmHg, p < 0.0001^14^.

When compare our results with the current evidence, the IOP lowering effect seemed to be slightly lower than that reported by Riss et al.^10^, Batlle et al.^11,13^, or Beckers et al.^14^, but similar to that found in the INN-005 study^18^. Differences in study protocols, concentration and placement of MMC, and the fact that the current study was conducted in a real setting, might justify such differences.

Moreover, the mean IOP reduction (30.6%) observed in the current study was in line with that estimated by Kudsieh et al.^19^. They estimated that for patients with a baseline IOP of 25 mmHg (higher than ours), the corresponding computed post-implant IOP with PMS would be 12 mmHg^19^.

Xen45 gel stent is a MIGS device that has been widely used. When compare the results of the current study with Xen45 studies, we can observe that PMS was comparable to Xen45 in lowering IOP or reducing the number of ocular hypotensive medications^20–29^.

The results of a head-to-head comparison between PMS (implanted alone) and PMS + Phaco found no differences in terms of IOP lowering between them.

In the current study, combining cataract surgery with PMS showed neither added IOP-lowering benefits nor greater reduction of ocular hypotensive medications.

The question of whether the best management of patients with cataract and glaucoma is performing cataract extraction followed by glaucoma surgery, or performing glaucoma surgery first followed by cataract, or performing a combined procedure remains.

Poelman et al.^30^ found not significant differences between Xen standalone or in combination with phacoemulsification in both IOP-lowering (32.6% and 20.9%, respectively, p = 0.054) and reduction of ocular hypotensive medications (66.9% and 63.8%, respectively, p = 0.638).

Similarly, the results of a meta-analysis comparing the efficacy and safety of Xen implant alone versus (vs) Xen + Phacoemulsification found no significant differences in IOP lowering between both strategies beyond month-3, although within week-1 Xen alone was mor effective^31^.

However, the results of another meta-analysis, which compared the efficacy of Xen45 alone and Xen45 + Phacoemulsification in open-angle glaucoma patients, found that Xen45 alone showed a greater IOP-lowering effect than Xen45-Phaco up to 6 months after surgery^32^.

Although RCT and real-world evidence can serve as a necessary complement to each other, the results are not always consistent. Besides differences in cohorts and conditions, which may justify the differences in results between RCT and real-world studies, other reasons, such us limited internal (real-world studies) or external (RCT) validity, missing data, etc. may justify these differences. Additionally, the difference between effectiveness (how the procedure works under real conditions) and efficacy (how the procedure works under ideal conditions) can help to explain the observed differences between RCT and real-world studies.

Regarding safety, the incidence of adverse events did not significantly differ from those previously published^12,14^. Adverse events were mild in severity and all were fully recovered with medical treatment.

The current study has some limitations that should be taken into account when interpreting its results. The first one is its retrospective design and the lack of a control group. Confounding factors and bias are inherent to retrospective studies. In order to minimize this limitation, we selected strict inclusion/exclusion criteria.

The second limitation has been not to calculate the sample size before starting the study. Although was not originally planned as a primary outcome in the study protocol, we conducted a post-hoc analysis comparing the IOP lowering effect between eyes who underwent PMS and those who underwent PMS + Phaco. The results of the current study suggested no differences between them. However, it might be underpowered for detecting such differences. In fact, with the observed pooled standard deviation and the number of eyes included in each group, the power for detecting a mean IOP lowering difference of 1.0 mmHg between PMS and PMS + Phaco groups, with an alfa error of 0.05 in a bilateral contrast, would be 13%. Therefore, further investigations with a larger number of patients recruited would be needed.

Despite these limitations, PMS, either alone or in combination with phacoemulsification, has demonstrated to be a valuable option for treating open-angle glaucoma patients. Further research is needed to investigate the potential risk factors associated with failure as well as role of MMC concentration on the clinical outcomes.

## Data Availability

The datasets generated during and/or analyzed during the current study are available from the corresponding author on reasonable request.
